# E-cadherin as a potential biomarker of malignant transformation in oral leukoplakia: a retrospective cohort study

**DOI:** 10.1186/1471-2407-14-972

**Published:** 2014-12-17

**Authors:** Sandra Ventorin von Zeidler, Talitha de Souza Botelho, Elismauro Francisco Mendonça, Aline Carvalho Batista

**Affiliations:** Department of Pathology, Federal University of Espírito Santo, Av. Marechal Campos, 1468 Maruípe, Vitória, ES Brazil ZIP Code 29.040-090; Department of Oral Pathology, Dental School, Federal University of Goiás, Goiânia, Goiás Brazil

**Keywords:** Oral leukoplakia, Oral cavity squamous cell carcinoma, E-cadherin, Epithelial dysplasia

## Abstract

**Background:**

Numerous attempts have been made to establish and develop tumor markers that could determine the susceptibility of normal tissues to transform into cancerous ones. To determine whether altered expression patterns of E-cadherin could be an early event in the progression of potentially malignant disorders to oral squamous cell carcinoma, this study aimed to assess the relationship between the immunoexpression of E-cadherin and the different degrees of epithelial dysplasia in oral leukoplakia.

**Methods:**

Surgically excised specimens from patients with oral leukoplakia (n = 31), oral cavity squamous cell carcinoma with cervical lymph node metastasis (n = 12) and normal oral mucosa (n = 9) were immunostained for E-cadherin. Oral leukoplakia samples were distributed into low and high risk group according to a binary system for grading oral epithelial dysplasia. Comparative analyses between E-cadherin expression and microscopic features (WHO histological grading and epithelial dysplasia) were performed by Pearson Chi-square test (*P* < 0.05).

**Results:**

Differences in E-cadherin expression were observed between normal oral mucosa and low risk oral leukoplakia (*P* = 0.006), low and high risk oral leukoplakia (*P* = 0.019), and high risk oral leukoplakia and oral cavity squamous cell carcinoma with cervical lymph node metastasis (*P* = 0.0001). In addition, as epithelia undergo dysplastic changes, the risk of malignant transformation increases, and there is a reduction or loss of E-cadherin expression by keratinocytes. Reduced E-cadherin expression was an early phenomenon and it was observed in moderate-severe dysplasia, showing that the loss of epithelial cohesion may be an indicator of progression to oral cavity squamous cell carcinoma.

**Conclusions:**

E-cadherin could be used as a novel biomarker to identify lesions with potential risk for malignant transformation, which may provide opportunities for prophylactic interventions in high risk patient groups.

## Background

Potentially malignant disorders include all clinical presentations that carry a higher risk of cancer when compared to healthy tissue. Much attention has been focused on oral leukoplakia (OL) due to its high incidence and potential for malignant transformation. The rates of malignant change vary based in part on the population, gender, tobacco habits and histological grading of dysplasia [1–3]. Oral epithelial dysplastic lesions may be morphological phenotypes of the different steps in the progression from normal to malignant tissue. Traditionally, oral epithelial dysplasia has been considered to be the progenitor for malignant changes [4]. The World Health Organization (WHO) grades oral epithelial dysplasia as mild, moderate or severe based on the importance of cellular atypia and the thickness of the dysplastic layers compared with the total epithelial height [5]. Therefore, the accurate assessment of the degree of dysplasia in oral lesions that potentially reflect malignant disease is quite challenging and creates barriers for the prediction and management of such conditions [6]. Furthermore, OL with similar histological phenotypes may present with different biological behavior. Nevertheless, the histopathological grading of epithelial dysplasia remains the one of the most clinically important predictors of the malignant potential of a lesion [1, 2, 7].

There have been numerous attempts to establish or even develop tumor markers to determine the susceptibility of normal tissues to transform into cancer [8–11]. Malignant transformation in many carcinomas is associated with the loss of epithelial phenotype and decreased differentiation. During this process, epithelial cells reorganize their cytoskeleton acquiring a mesenchymal phenotype, a process known as epithelial to mesenchymal transition (EMT). The expression of mesenchymal genes is often accompanied by loss of epithelial characteristics, including reduced inter-cellular adhesion, loss of epithelial cell polarity and increased motility. These EMT features are seen in oral epithelial dysplasia and in their progression to cancer [12, 13]. Cell adhesion molecules play more than a purely structural role within stratified squamous epithelia. There is a strong relationship between reduced expression of these adhesion molecules, decreased differentiation and increased invasiveness [8, 10, 14–18].

The cadherins are a family of homophilic cell adhesion proteins that are expressed in a variety of tissues and require Ca^2+^ binding for their adhesiveness, rigidity and stability. E-cadherin is a cell membrane-associated protein involved in cell-cell adhesion, and it is generally localized to the lateral surfaces of epithelial cells in a region of cell-cell contact that is known as the adherens junction. As an intercellular adhesion molecule, E-cadherin plays an important role in establishing and maintaining intercellular connections and by directly eliciting signals involved in tissue morphogenesis for epithelial integrity. E-cadherin is the key player in inducing cell polarity and organizing the epithelium. The exact mechanism that normal oral epithelium becomes a dysplastic tissue is not known. However, it has been shown that E-cadherin mediated-cell-cell adhesion can regulate important cell signaling pathways influencing mechanisms of proliferation, differentiation, as well as apoptosis [19, 20]. Moreover, E-cadherin’s intracellular ligand β-catenin regulates Wnt-signaling pathway acting as transcriptional activator involved in tumorigenesis; whereas in parallel, it mediates the cadherin/catenin complex interaction with epidermal growth factor receptor [21]. Further, dysfunctional E-cadherin-mediated cell adhesion has been suggested to be associated with the loss of differentiation and acquisition of an invasive phenotype and may be used as a potential biomarker of tissue transformation [22, 23].

In most cancers of epithelial origin, including head and neck squamous cell carcinoma, E-cadherin-mediated cell-cell adhesion is lost concomitantly with progression towards tumor malignancy [20, 24–26]. Although some studies have explored the immunoreactivity of E-cadherin in potentially malignant disorders, non-tumor epithelium adjacent to oral cancer and oral squamous cell carcinoma, it remains unclear if E-cadherin could be used as biomarker to predict malignant transformation [10, 22, 23]. Nevertheless, there have been few reports on the immunohistochemical expression of this protein in oral precancerous lesions with respect to dysplasia grade. In addition, there is limited data on the usefulness of E-cadherin for estimating the risk of developing tumors associated with the progression of the dysplasia-carcinoma sequence in the oral cavity and on the potential for this molecule to mediate a signaling pathway driving oral squamous cell carcinoma growth and invasion.

To determine whether altered expression patterns of E-cadherin could be an early event in the progression of potentially malignant disorders to oral squamous cell carcinoma and invasiveness, this study aimed to assess the relationship between the immunoexpression of E-cadherin and the different degrees of epithelial dysplasia in OL.

## Methods

### Patients and clinical specimens

The samples from this retrospective cohort study consisted of surgically excised specimens from 31 patients with a clinical diagnosis of OL who were periodically monitored at Oral Disease Center of Goiás of the Federal University of Goiás, Brazil. Specimens from 12 oral cavity squamous cell carcinoma with cervical lymph node metastasis (OCSCC N^+^) and 9 individuals with normal oral mucosa (control) were also included to compare the pattern of E-cadherin expression. All specimens were retrieved from the archives of the Oral Pathology Laboratory of the Dental School at the Federal University of Goiás, Brazil. Clinical data (i.e., gender, age, ethnic group, tobacco and alcohol consumption, lesion/tumor location and lesion/tumor size) were obtained from medical records.

The inclusion criteria during this study were patients of both sexes, over 30 years old, submitted for the surgical treatment, T2 ⁄ T3 size of the primary tumor or those with clinical diagnostic of OL. The exclusion criteria were patients with squamous cell carcinoma in other sites and those who received radiotherapy, chemotherapy or any other treatment prior to surgery.

This study was approved by the Institutional Ethics Committee for Human Subjects of the Araujo Jorge Hospital, Goiás Combat Cancer Association (Protocol 2010–015). The experiments were undertaken with the understanding and written consent of each subject according to the ethical principles.

### Light microscopy

All specimens were fixed in 10% buffered formalin (pH 7.4) and were then paraffin embedded. The microscopic features of the samples were evaluated from the analysis of one 5-μm section of each sample, which was stained with hematoxylin and eosin. Epithelial dysplasia was graded according to the WHO classification (2005) [5]. Using a binary system for grading oral epithelial dysplasia, OL were distributed into a low risk (observation of less than four architectural changes or less than five cytological changes) and high risk (based on observing at least four architectural changes and five cytological changes) group [27]. The architecture features noted were as follows: irregular epithelial stratification, loss of basal cell polarity, drop-shaped rete ridges, increased number of mitotic figures, abnormally superficial mitoses, premature keratinization in single cells and keratin pearls within rete ridges. The cytology changes included the following: abnormal variation in nuclear and cellular size and shape, increased nuclear-cytoplasmic ratio, increased nuclear size, atypical mitotic figures, increased number and size of nucleoli and hyperchromatism. All of the OCSCC sections were graded according to the WHO classification of tumors [5].

### Immunohistochemistry

Paraffin-embedded tissues were sectioned (3 μm) and collected in series on glass slides coated with 2% 3-aminopropyltriethsilane (Sigma-Aldrich, St Louis, MO, USA). Following deparaffinization by immersion in xylene, the sections were immersed in alcohol and incubated with 3% hydrogen peroxide for 40 min. To retrieve antigens, the sections were immersed in citrate buffer (pH 6.0) for 20 min. Afterwards, the sections were incubated for 20 min with 3% normal goat serum at room temperature. The slides were incubated at 4°C overnight with the primary antibody monoclonal mouse anti-E-Cadherin human (SPM471, Spring Bioscience, Pleasanton, CA, USA) at 1:200 in a humidifier. After washing with Phophate Buffered Saline, the sections were labeled with TrekAvidin-HRP Label (STHRP700 L10; Biocare Medical, Concord, CA, USA) and then incubated with 3,3’-diaminobenzidine (K3468; DAKO, Glostrup, Denmark) for 2–5 min at room temperature. The sections were then stained with Mayer’s hematoxylin and covered (Entellan-Mikroskopie-Merck, Darmstadt, Germany). Negative controls were obtained through the omission of primary antibody, and normal oral mucosa samples with known positive reactivity were included as positive controls.

### Cell counting and statistical analysis

The analysis of E-cadherin immunoexpression was carried taking in count the percentage of positive staining cells in relation to the whole examined area. A semi-quantitative scoring system was used, which was based on the characteristic staining pattern on a four-point scale: 0, negative with absent or discontinued membranous staining; 1, weak with 1-50% of cell staining; 2, moderate with 51-75% of cell staining and 3, strong with more than 75% of cells staining. A total index score was obtained by adding the results of all layers (basal, parabasal and keratinized) in the groups with normal oral mucosa and OL; tumor front was analyzed at OCSCC N^+^ group. Epithelial cells were considered positive if they had the evidence of brown membranous staining. Cell counts were performed by one investigator in ten alternate microscopic high-power fields (x400) using an integration graticule (Standard 20 ZEISS; Carl Zeiss, Gottingen, Germany).

Comparative analyses between E-cadherin expression and microscopic features (WHO histological grading and epithelial dysplasia) were performed using the Pearson Chi-square test. The level of statistical significance was accepted at *P* < 0.05.

## Results

The cases analyzed were distributed into the following groups: OL (n = 31), OCSCC N^+^ (n = 12) and control (n = 9), and the mean age of the groups was 50.9 years (CI = 31–79), 56.4 years (CI = 42–80) and 20.6 years (CI = 17–27), respectively. Patients with oral leukoplakia were monitored in a period, which ranged from 6–18 months (mean 12.4 months), and during this period malignant transformation was not observed. All OCSCC cases had T2/T3 staging, and the presence of lymph node metastasis was confirmed microscopically. The other features of our series are summarized in Table 1.

**Table 1:** **Main clinical findings of patients with OL (n = 31), OCSCC N** ^**+**^ **(n = 12) and controls (n = 9)**

***Clinical features***	***OL (%)***	***OCSCC N*** ^***+***^ ***(%)***	***Controls (%)***
**Gender**			
Male	45.2	83.3	22.2
Female	54.8	16.7	77.8
**Tobacco**			
Yes	80.6	100	0
No	19.4	0	100
**Alcohol**			
Yes	41.9	66.6	0
No	58.1	33.4	100
**Anatomic site**			
Buccal mucosa	58.1	0	88.9
Oral tongue	41.9	100	11.1

*OL*, oral leukoplakia; *OCSCC N* ^*+*^, oral cavity squamous cell carcinoma with cervical lymph node metastasis.

After microscopic evaluation, OL samples were classified using the binary system [27]. Low risk corresponded to cases of OL without dysplasia or mild dysplasia (n = 23), while 8 cases were classified as high risk and corresponded to cases of OL with moderate or severe epithelial dysplasia. All OCSCC cases were moderately or poorly differentiated (grades II-III).

**Figure 1:**
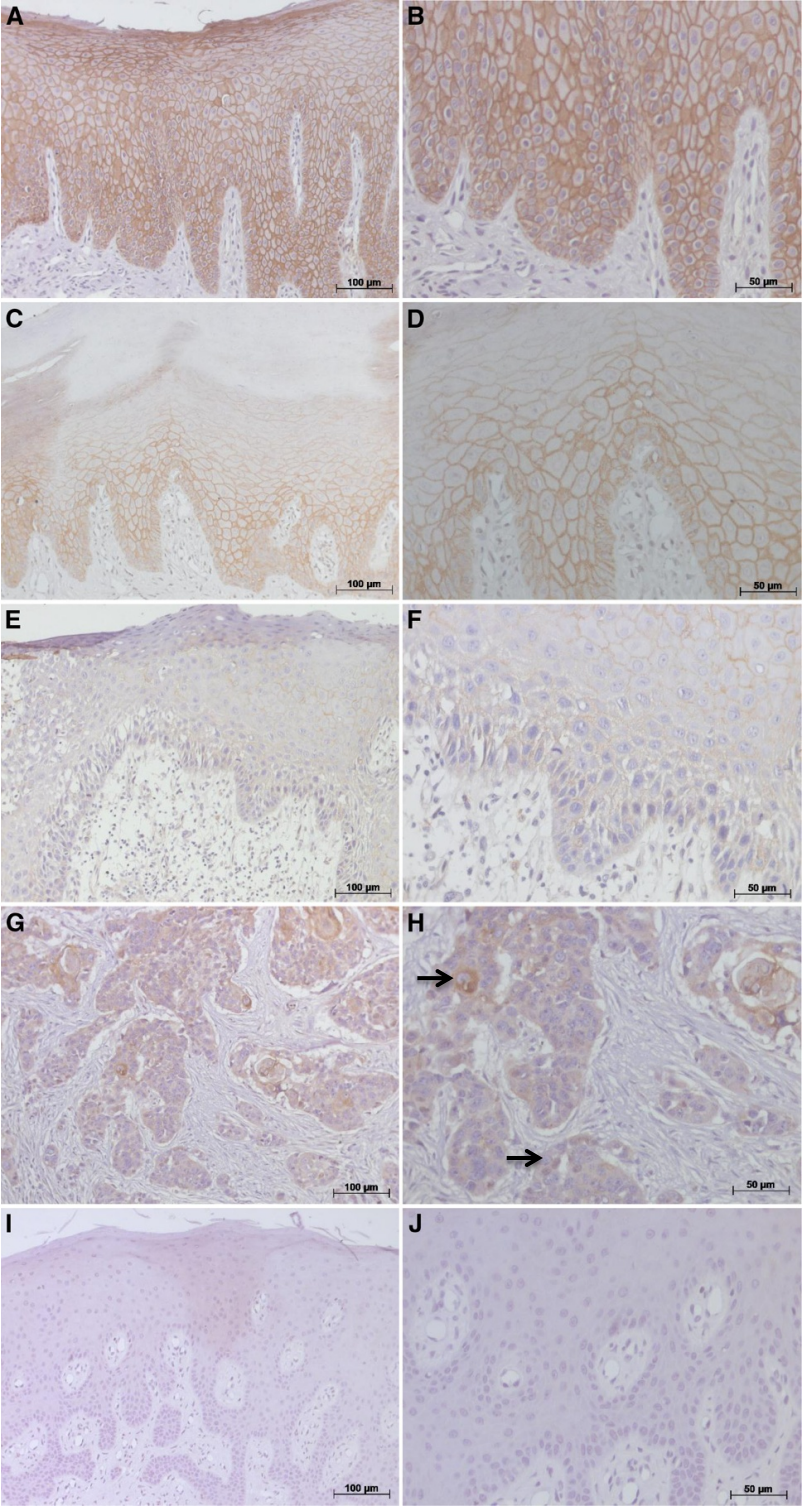
**Immunohistochemical E-cadherin expression in oral normal mucosa, oral leukoplakia and oral cavity squamous cell carcinoma. (A, B)** intense membranous E-cadherin expression in the basal and parabasal layers in normal oral mucosa; **(C, D)** show reduced E-cadherin expression in the parabasal layer in low risk oral leukoplakia; **(E, F)** illustrate the decrease in E-cadherin expression in all oral epithelial layers in high risk oral leukoplakia; **(G, H)** show a moderately differentiated OCSCC with loss of E-cadherin expression in the cell membrane of the neoplastic cells in the tumor invasion front; cytoplasmic and nuclear staining were observed (arrow); **(I, J)** Negative control. Immunohistochemical staining, original magnifications; x200 **(A, C, E, G, I)** and x400 **(B, D, F, H, J)**.

Differences in the E-cadherin expression were observed between all groups (*P* = 0.0001). In normal oral mucosa E-cadherin immunohistochemical staining was observed predominantly in the basal and parabasal layers, showing a continuous and homogeneous staining, while marking was discontinuous or absent in the upper third of the oral epithelium (Figure 1A-B). No labeling was observed in the cytoplasm or keratinized layer. Semi-quantitative analysis revealed a score of 3 in the labeled cells of the control group (Table 2).

**Table 2:** **Percentage of samples per positivity of E-cadherin immunoexpression**

		***Scoring positivity***				***Pearson Chi-square*** *ǂ*
	***Groups***	***0***	***1***	***2***	***3***	***p-value***
***E-cadherin expression in epithelial cells*** ***(% of samples)***	Control	-	-	-	100	0.006*
	LR OL	-	-	52.18	47.82	
	LR OL	-	-	52.18	47.82	0.019*
	HR OL	-	12.50	87.50	-	
	HR OL	-	12.50	87.50	-	0.0001*
	OCSCC N^+^	33.34	66.66	-	-	

*ǂPearson Chi-square test*, *P* = 0.0001.

*Represents a statistically significant difference within the paired groups (*Pearson Chi-square test*). *LR OL,* low risk oral leukoplakia*; HR OL,* high risk oral leukoplakia*; OCSCC N* ^*+*^ *,* oral cavity squamous cell carcinoma with cervical lymph node metastasis.

In the low risk OL group (n = 23), a reduction in the E-cadherin expression was observed mainly in the parabasal layer when compared to the normal oral mucosa (*P* = 0.006) (Figure 1C-D). Through semi-quantitative analysis scores 2 and 3 were obtained (Table 2). However, in the high risk OL group (n = 8), the E-cadherin expression was reduced in all epithelial layers (Figure 1E-F). Scores 1 and 2 were obtained from semi-quantitative analysis and a significant reduction in E-cadherin expression compared to the low risk OL group (*P* = 0.019) was observed (Table 2).

In the OCSCC N^+^ group (n = 12), there was a reduction in E-cadherin expression in the cell membrane of the neoplastic cells in the tumor invasion front. In addition, cytoplasmic and nuclear staining was noted (Figure 1G-H). Scores 0 and 1 were obtained in the OCSCC N^+^ group (Table 2). It is noteworthy that strong staining of keratin pearls was found and not considered to be positive staining. A positive E-cadherin score was noted for all groups listed in Table 2.

## Discussion

The presence and severity of dysplasia in a potentially malignant oral lesion is currently the standard in predicting the risk of malignant transformation [3, 4]. Studies have noted that pre-cancerous lesions with epithelial dysplasia develop into cancer more readily than lesions without such dysplasia [1, 11], while others have shown that dysplasia regressed with time [7]. Malignant transformation may also take place in non-dysplastic leukoplakia, and there is currently no information available in the literature considering differences in the behavior or risk of malignant transformation [2, 6, 7].

Malignant transformation in many carcinomas is associated with the loss of epithelial differentiation and a gain in a mesenchymal phenotype, which has been described in events associated with embryogenesis, healing and metastasis [13]. Recent studies suggest that epithelial to mesenchymal transition may be a predictor of OCSCC progression and prognosis [8, 12]. The expression of mesenchymal genes with carcinoma progression is often accompanied by an increase in cell motility and the loss of epithelial features, including reduced intercellular adhesion and a loss of epithelial cell polarity. These features are observed in cases of not only OCSCC progression but also oral epithelial dysplasia. These changes may be found early in the development of OCSCC, and the identification of genes and their products that play a role in the transition process may be potential biomarkers of malignant transformation [8, 9]. One of the functions of E-cadherin appears to be the control of cell motility during embryogenesis or tissue healing, with the downregulation of E-cadherin allowing for migration of regenerating epithelium over the area of ulceration [19]. This role in the control of cell motility has led to the suggestion that E-cadherin is an 'invasion suppressor’ molecule and that in carcinogenesis, loss of E-cadherin permits or enhances the invasion of adjacent normal tissue [10, 20].

This study used immunohistochemistry to quantify and analyze expression patterns of E-cadherin in normal oral mucosa, oral epithelial dysplasia and OCSCC N^+^ to investigate the role of this molecule in oral carcinogenesis and its ability to predict transformation in potentially malignant lesions. Normal oral mucosa was used as a positive control, showing strong expression of E-cadherin particularly in basal and parabasal layers. This suggests an important role of E-cadherin in the unchanged histopathophysiology and maintenance of epithelial tissue structure [22]. The expression of E-cadherin in the low risk OL group was similar to the control group, as also described by Williams et al. (1998) [23]. Our results showed greater loss or interruption of the adhesion molecule E-cadherin in the cell membrane in high risk OL groups and OCSCC N^+^ compared to low risk OL groups and normal oral mucosa (Table 2). Furthermore, the reduction in E-cadherin expression with increasing severity of histopathological dysplasia grading was also observed. Previous studies have found E-cadherin expression to be significantly reduced in dysplastic oral mucosa and OCSCC [8, 15, 17, 18], considering that loss of cohesion is one of the key features of dysplasia. Also recently published data by Freitas Silva et al. [21] reinforced our findings by demonstrating E-cadherin downregulation leading to phenotypic changes in early stages of oral carcinogenesis. It is believed that the E-cadherin downregulation is the central event of EMT, as it promotes loss of cell-cell contacts as a key step during cancer progression and metastasis allowing the neoplastic cells to move through the extracellular matrix. The loss of E-cadherin function during tumor progression can be caused by various genetic or epigenetic mechanisms. In most cases, E-cadherin expression is downregulated at the transcriptional level [20]. As a direct consequence of transcriptional inactivation, the E-cadherin gene locus is epigenetically silenced by hypermethylation, leading to further downregulation of E-cadherin expression. Additionally, there are some evidence that both Twist and p-Akt were associated with E-cadherin downregulation and loss of cell-to-cell adhesion in EMT [21]. Our results also show that in a high percentage of samples from OCSCC N^+^, loss of E-cadherin expression in the membrane was associated with the cytoplasmic and nuclear expression of this protein by neoplastic cells. While other authors have also shown similar results [16, 25], the intensity and frequency of the cytoplasmic expression of E-cadherin by neoplastic cells remains controversial [23, 28]. Therefore, additional studies are needed in order to clarify the mechanisms by which the reduction and loss of E-cadherin expression during malignant transformation occurs.

In addition, reduced expression of E-cadherin is associated with carcinomas that have a striking infiltration growth pattern, show poor differentiation, develop metastases and carry a poor prognosis [14, 16, 24, 26, 29]. Thus, analyzing moderately and poorly differentiated OCSCC with nodal involvement reduced membranous E-cadherin expression was observed, along with high cytoplasmic E-cadherin expression and eventual nuclear staining (Figure 1G-H). Loss of E-cadherin function elicits active signals that support tumor-cell migration, invasion and metastatic dissemination and it may be involved in the infiltrative process and nodal involvement. Another study also observed that increased cytoplasmic E-cadherin was associated with clinical and nodal stage [16]. Furthermore, altered E-cadherin expression has been related to metastases, as it favors cell locomotion, proteolysis, survival and proliferation in primary and distant sites [30]. However, some studies have not observed a correlation between the pattern of staining and the histological grade of the tumor, suggesting that loss of E-cadherin is not necessary for the acquisition of the malignant phenotype [10]. These data were supported by in *vitro* studies where an increased infiltrative or invasive potential was associated with low levels of E-cadherin expression, while non-invasive but still malignant lines showed high E-cadherin expression levels [24]. It is possible that E-cadherin could be present but non-functional. Further studies should concentrate on the role of E-cadherin in modulating the behavior of cells*.*

## Conclusions

In summary, reduced E-cadherin expression was an early phenomenon, as we observed it in moderate-severe dysplasia, suggesting that loss of epithelial cohesion may be an indicator of possible evolution. Further, as epithelia undergo dysplastic changes and the risk of malignant transformation increases, there is a reduction in or loss of E-cadherin expression by keratinocytes. Therefore, E-cadherin could be used as a novel biomarker to identify OL lesions at increased risk for transformation, which may provide opportunities for prophylactic intervention in high risk patient groups.

## Abbreviations

OL: Oral leukoplakia
WHO: World Health Organization
OCSCC: Oral cavity squamous cell carcinoma
OCSCC N^+^: Oral cavity squamous cell carcinoma with cervical lymph node metastasis.
